# Supplementary material for: LSD1 protects against hippocampal and cortical neurodegeneration
Source: Nat Commun. 2017 Oct 9;8:805. doi: 10.1038/s41467-017-00922-9 (PMC5634471; doi:10.1038/s41467-017-00922-9)
Supplement: Supplementary file 1 — Supplementary Information [file 41467_2017_922_MOESM1_ESM.pdf]

Description of Supplementary Files

File Name: Supplementary Information
Description: Supplementary Figures and Supplementary Table

File Name: Supplementary Movie 1
Description: *Lsd1*^{CAGG} motor defect. (a,b) (0:00-0:24) Normal walking, posture and hindlimb extension by tamoxifen injected *Cre* minus control mice. (c,d) (0:25-0:47) Onset of moderate hindlimb clasping phenotype while retaining walking ability and maintaining posture in *Lsd1*^{CAGG} mice. (e) (0:48-1:09) Progression of *Lsd1*^{CAGG} phenotype shown by increase in hindlimb clasping severity and docile behavior while maintaining posture. (f,g) (1:10-1:59) Progression of phenotype to severe hindlimb clasping, docile behavior, lack of maintaining posture and lack of coordination in *Lsd1*^{CAGG} mice. (h) (2:00-2:32) Example of severe hindlimb clasping, inability to keep eyes open, lack of grooming and failure to use hindlimbs in *Lsd1*^{CAGG} mouse. (i,j) (2:33-3:23) Examples of failure to maintain posture when placed on side in *Lsd1*^{CAGG} mice. (k-m) (3:24-4:42) Terminal phenotype with no movement, hindlimb and forelimb clasping, and complete failure to maintain posture in *Lsd1*^{CAGG} mice.

File Name: Supplementary Movie 2
Description: Morris Water Maze Probe Trial. (a,b) Video of representative probe trial of Morris water maze taken on Day 6 after five day training period of control (a) and *Lsd1*^{CAGG} (b) mice, displaying capable swimming ability and lack of contextual learning capacity in *Lsd1*^{CAGG} mice. Position of the platform during the five day training that has been removed for the probe trial is represented by a red circle.

File Name: Supplementary Data 1
Description: Expression changes in *Lsd1*^{CAGG} hippocampus**.** Spreadsheets for all, significantly upregulated, and significantly downregulated transcripts. Provided for each genotype with two biological replicates are the Reads Per Kilobase per Million (RPKM) values, $\log_2$ fold change, *P*-value, and q-value as determined by CuffDiff. Samples are two tamoxifen injected *Cre* minus control hippocampi (control) and two *Lsd1*^{CAGG} terminal hippocampi (cre).

File Name: Peer Review File

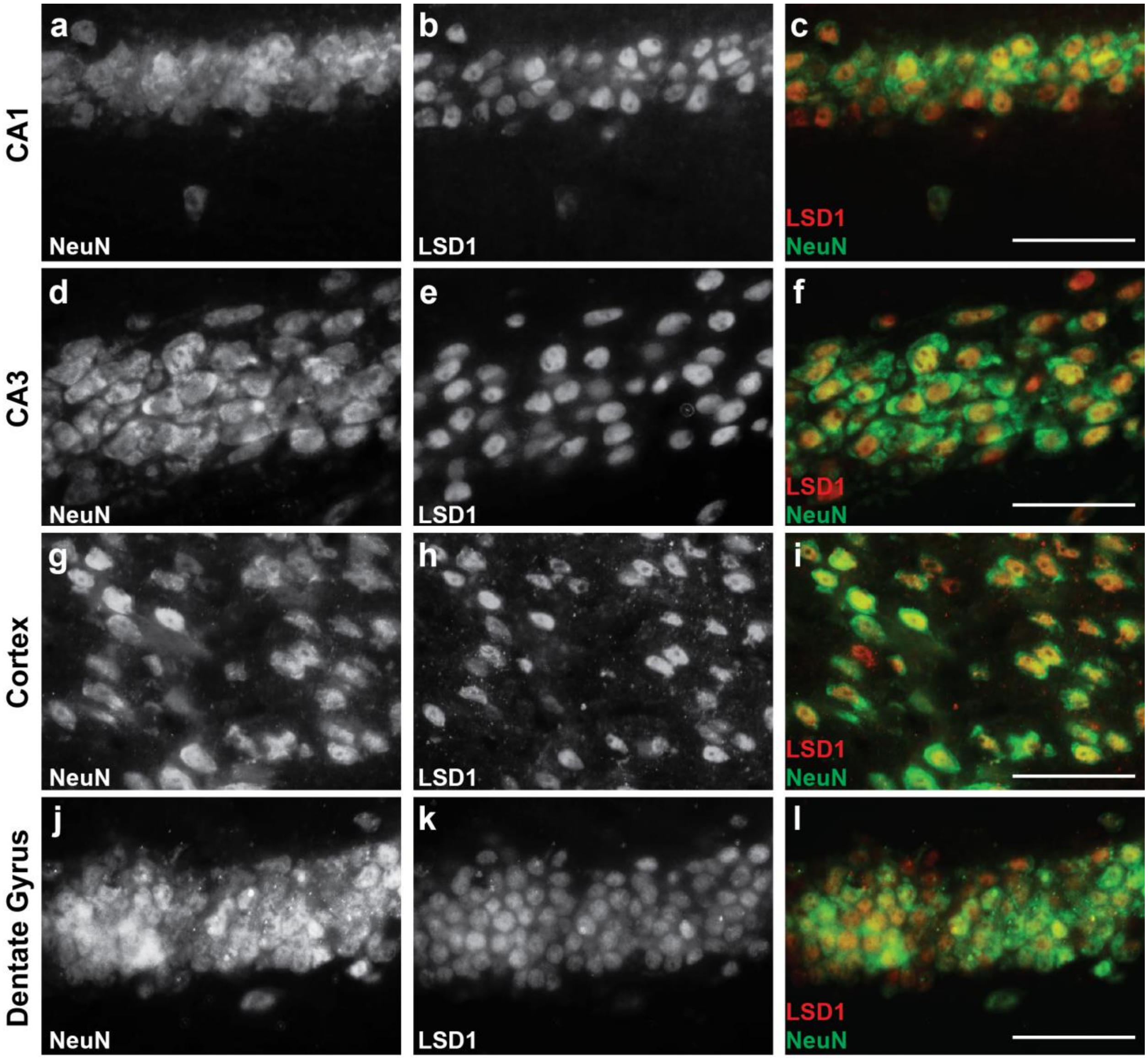

Supplementary Figure 1 | LSD1 expression in adult murine hippocampal and cortical neurons.

(**a-l**) Immunofluorescence labelling with the neuronal nucleus marker NeuN (**a,d,g,j**,), LSD1 (**b,e,h,k**,) and merged (**c,f,i,l**) showing LSD1 protein in neurons of the CA1 (**a-c**) and CA3 (**d-f**) of the hippocampus, cortex (**g-i**) and dentate gyrus (**j-l**) of wild-type mice. Scale bars= 50µm.

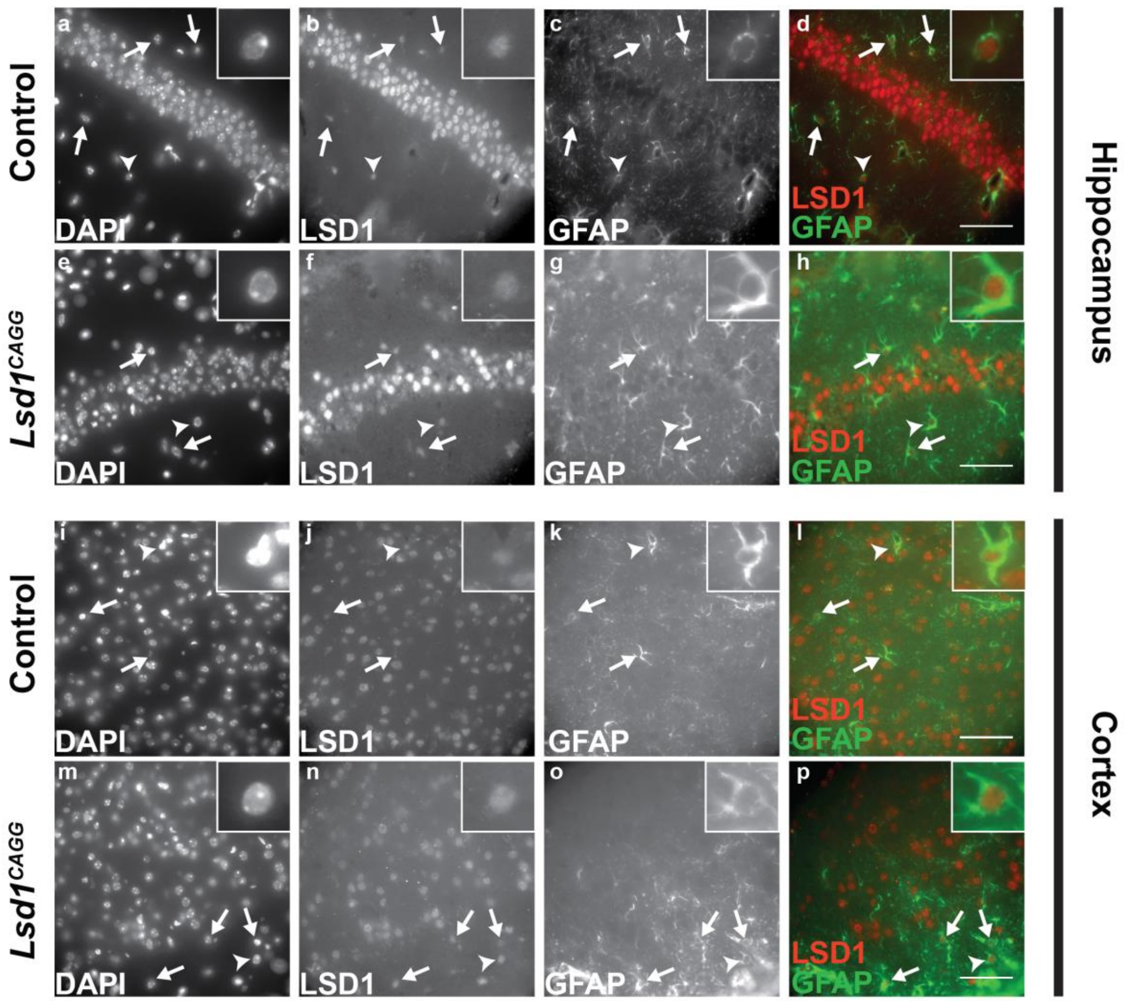

Supplementary Figure 2 | LSD1 expression in adult murine hippocampal and cortical astrocytes.

(a-p) Immunofluorescence labeling of DAPI (**a,e,i,m**), LSD1 (**b,f,j,n**), GFAP (**c,g,k,o**) and LSD1/GFAP merge (**d,h,l,p**) showing LSD1 is present in GFAP positive astrocytes both in control hippocampus **(a-d)** and cortex **(i-l),** as well as *Lsd1*^{CAGG} hippocampus (**e-h**) and cortex **(m-p)**, indicating LSD1 expression is not affected in astrocytes. Arrows denote representative nuclei. Arrowheads indicate nuclei magnified in inset. Scale bars= 50μm.

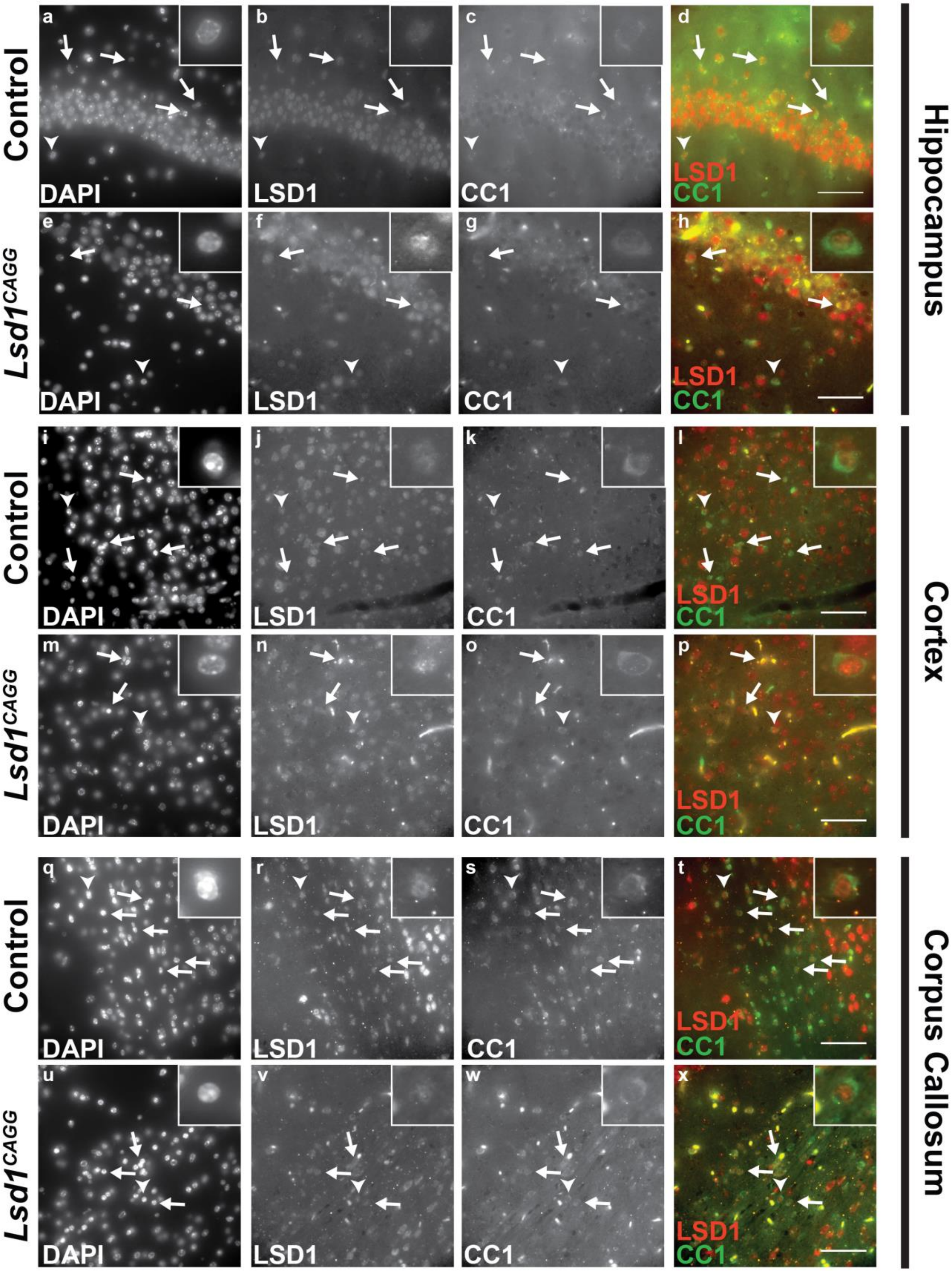

Hippocampus
Cortex
Corpus Callosum
Control
Lsd1CAGG
a
b
c
d
e
f
g
h
i
j
k
l
m
n
o
p
q
r
s
t
u
v
w
x
DAPI
LSD1
CC1

Supplementary Figure 3 | LSD1 expression in adult murine hippocampal and cortical oligodendrocytes.

(a-x) Immunofluorescence labeling of DAPI (**a,e,i,m,q,u**), LSD1 (**b,f,j,n,r,v**), CC1 (**c,g,k,o,s,w**) and LSD1/CC1 merge (**d,h,l,p,t,x**) showing LSD1 is present in CC1 positive oligodendrocytes in the control hippocampus **(a-d)**, cortex **(i-l)**, and corpus callosum **(q-t)**, as well as $\textit{Lsd1}^{CAGG}$ hippocampus (**e-h**), cortex **(m-p)**, and corpus callosum **(u-x)**, indicating LSD1 expression is not affected in oligodendrocytes. Arrows denote representative nuclei. Arrowheads indicate nuclei magnified in inset. Scale bars= 50μm.

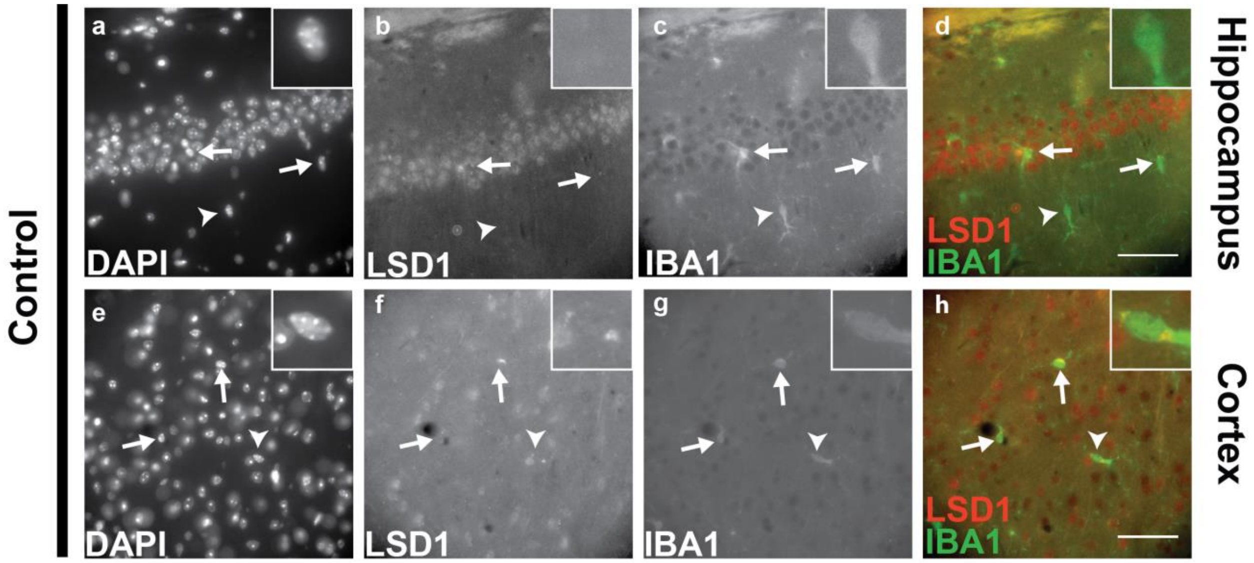

Supplementary Figure 4 | LSD1 is not expressed in adult murine hippocampal and cortical microglia.

(a-h) Immunofluorescence labeling of DAPI (**a,e,i,m**), LSD1 (**b,f,j,n**), IBA1 (**c,g,k,o**) and LSD1/IBA1 merge (**d,h,l,p**) showing LSD1 is absent in IBA1 positive microglia in the hippocampus **(a-d)** and cortex **(e-h)** of control mice. Arrows denote representative nuclei. Arrowheads indicate nuclei magnified in inset. Scale bars= 50µm.

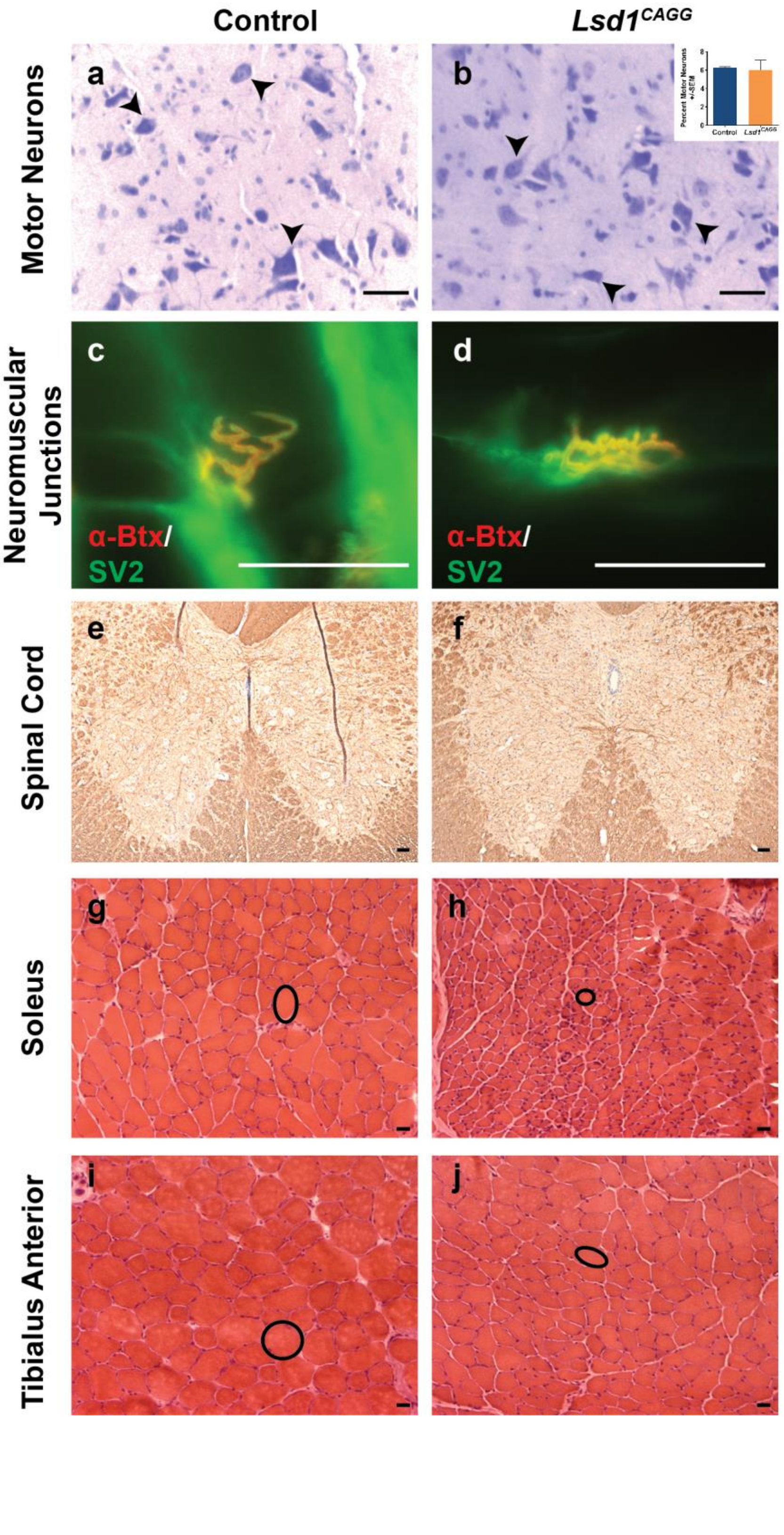
Control
Lsd1CAGG
Motor Neurons
Neuromuscular Junctions
Spinal Cord
Soleus
Tibialus Anterior
a
b
c
d
e
f
g
h
i
j
α-Btx/
SV2
Percent Motor Neurons +/-SEM
Control
Lsd1CAGG

Supplementary Figure 5 | Absence of spinal cord motor neuron and muscle defects in *Lsd1^{CAGG}* Mice.

(**a,b**) Thionin staining of control (**a**) and *Lsd1^{CAGG}* (**b**) ventral horn spinal cord motor neurons (arrowheads). Inset shows histogram of percentage motor neurons (per total ventral horn nuclei) for control (n = 3) and *Lsd1^{CAGG}* (n = 4). Values represent mean ± s.e.m. No significant difference between genotypes (p = 0.838, unpaired t test) (**c,d**) Immunofluorescence of neuromuscular junctions showing SV2 (presynaptic motor neurons, green) and fluorescent α-bungarotoxin (muscle acetylcholine receptors, red) in control (**c**) and *Lsd1^{CAGG}* (**d**). Co-localization SV2 and α-btx demonstrate an intact junction. **(e,f**) Immunohistochemistry (IHC) of myelin basic protein (MBP) in lower cervical spinal cord showing no difference in myelin amount or distribution between control **(e)** and *Lsd1^{CAGG}* **(f)**. IHC is counterstained with hematoxylin. (**g-j**) H&E staining of soleus (**g,h**) and tibialus anterior muscles (**i,j**) showing muscle fiber size (circles) in controls (**g,i**) compared to reduced cell size in *Lsd1^{CAGG}* (**h,j**). Absence of gaps in the tissue and absence of centrally located nuclei indicate a lack of muscle degeneration. Scale bars= 50µm.

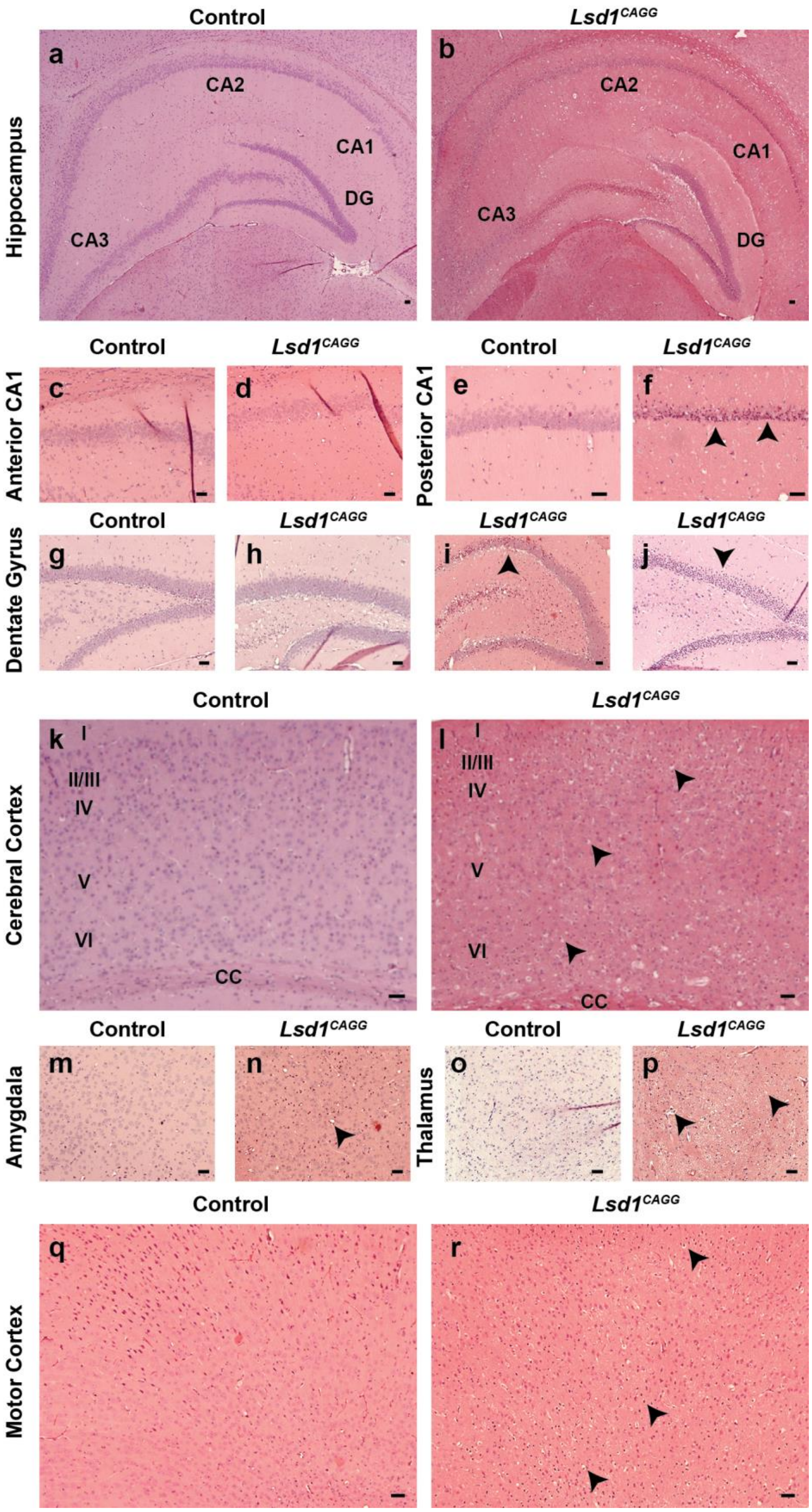

Control
Lsd1CAGG
Hippocampus
a
CA2
CA1
DG
CA3
b
CA2
CA1
CA3
DG
Control
Lsd1CAGG
Control
Lsd1CAGG
Anterior CA1
c
d
Posterior CA1
e
f
Control
Lsd1CAGG
Lsd1CAGG
Lsd1CAGG
Dentate Gyrus
g
h
i
j
Control
Lsd1CAGG
Cerebral Cortex
k
I
II/III
IV
V
VI
CC
l
I
II/III
IV
V
VI
CC
Control
Lsd1CAGG
Control
Lsd1CAGG
Amygdala
m
n
Thalamus
o
p
Control
Lsd1CAGG
Motor Cortex
q
r

Supplementary Figure 6 | Neurodegeneration in *Lsd1^{CAGG}* mice.

(**a-r**) H&E staining of control and *Lsd1^{CAGG}* hippocampus (**a,b**), anterior and posterior CA1 (**c-f**), dentate gyrus (**g-j**), cerebral cortex (**k,l**), amygdala (**m,n**), thalamus (**o,p**), and motor cortex (**q,r**). (**a,b**) Distribution of pyknosis in *Lsd1^{CAGG}* hippocampus with CA1 being most affected, and CA2 and CA3 moderately affected (**b**), compared to control with no pyknosis (**a**). (**c-f**) Increasing severity of pyknosis from anterior (**d**) to posterior (**f**) from the same *Lsd1^{CAGG}* hippocampus compared to control with no pyknosis (**c,e**). (**g-j**) Varying severity of pyknosis from three *Lsd1^{CAGG}* dentate gyruses; unaffected (**h**), moderately affected (**i**) completely affected (**j**) compared to control with no pyknosis (**g**). (**k,l**) Distribution of pyknosis in cerebral cortex of *Lsd1^{CAGG}* (**l**) in layers II/III, IV and VI, compared to control with no pyknosis (**k**), CC designates corpus callosum. (**m-r**) Distribution of pyknosis in the amygdala (**n**), thalamus (**p**) and motor cortex (**r**) of *Lsd1^{CAGG}* compared to control of same brain regions with no pyknosis (**m,o,q**). Arrowheads denote pyknotic nuclei. Scale bars= 50µm.

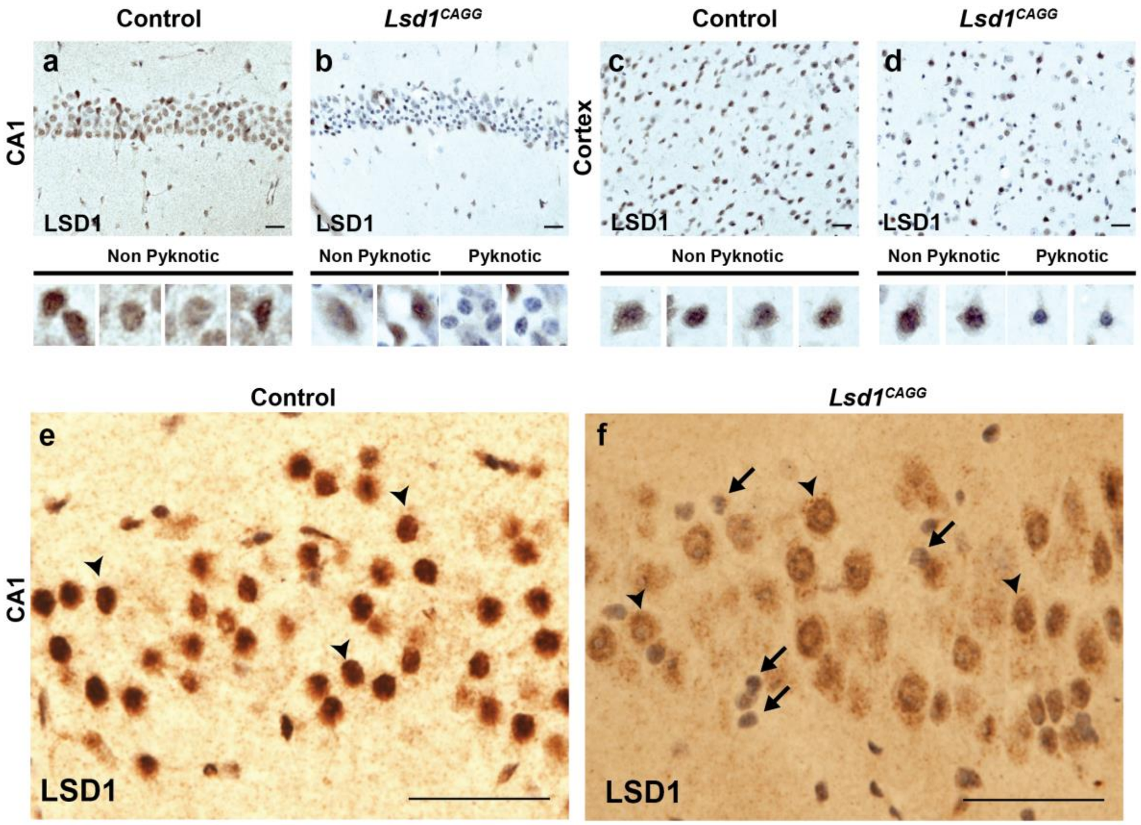

Supplementary Figure 7 | LSD1 in different cell types

(**a-d**) LSD immunohistochemistry (IHC) in control (**a,c**) and *Lsd1*^{CAGG} (**b,d**) CA1 (**a,b**) and cerebral cortex (**c,d**) showing the breadth of pyknosis associated with absence of LSD1 immunoreactivity in *Lsd1*^{CAGG} (**b,d**) compared to control (**a,c**) where LSD1 immunoreactivity is ubiquitous and pyknosis is absent. Images are the source images from Figure 1a-d. Insets below highlight immunoreactive non-pyknotic neuronal nuclei and non-immunoreactive pyknotic neuronal nuclei. (**e,f**) LSD1 immunohistochemistry in control (**e**) and *Lsd1*^{CAGG} (**f**) CA1 ten weeks after a single, reduced dose of tamoxifen (1 mg/ 40g of body mass). Every neuronal nucleus in control and most neuronal nuclei in *Lsd1*^{CAGG} mice display normal LSD1 immunoreactivity and are not pyknotic (arrowheads). However, a small number of nuclei are non-immunoreactive for LSD1 and are also pyknotic (arrows), which is consistent with a cell

autonomous effect on neuronal cell death.**)** All IHC **(a-f)** is counter stained with hematoxylin.

Scale bars= 50µm.

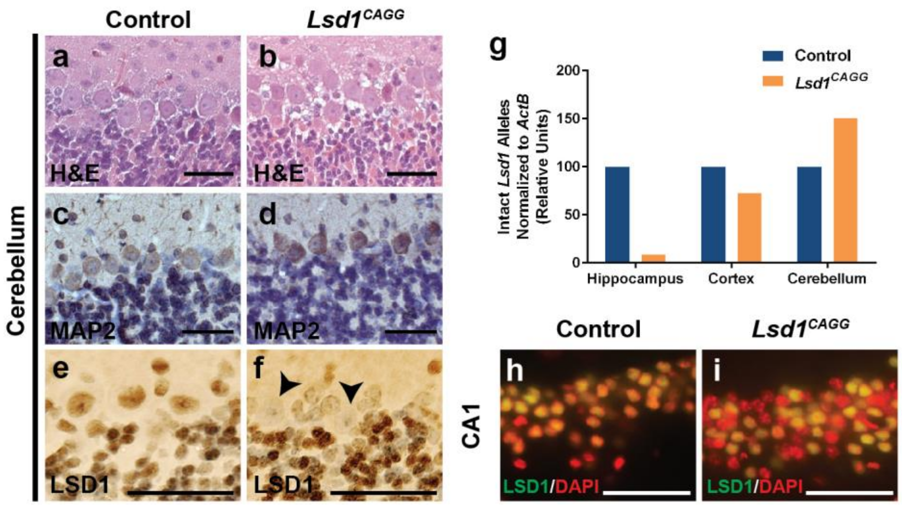

Supplementary Figure 8 | Absence of neurodegeneration in the *Lsd1^{CAGG}* cerebellum.

(**a,b**) H&E staining of control (**a**) and *Lsd1^{CAGG}* (**b**) cerebellum showing similar cellular morphology and lack of pyknotic nuclei in *Lsd1^{CAGG}*. (**c,d**) MAP2 immunohistochemistry (IHC) in control (**c**) and *Lsd1^{CAGG}* (**d**) showing similar distribution in cerebellar neurons. (**e,f**) LSD1 IHC in control (**e**) and *Lsd1^{CAGG}* (**f**) cerebellum showing lack of LSD1 in some (arrowheads), but not all *Lsd1^{CAGG}* purkinje neurons. (**g**) Quantification of intact *Lsd1* alleles (revealing the extent of *Lsd1* deletion) in control (blue) and *Lsd1^{CAGG}* (orange) hippocampus 24 hours after tamoxifen injection, and in cortex and cerebellum at terminal phenotype. Data are shown as relative units normalized to *ActB*, where the control value is set to 100. (**h,i**) Merge of LSD1 (green) immunofluorescence and DAPI (red) in control (**h**) and *Lsd1^{CAGG}* (**i**) CA1 nuclei showing LSD1 protein remaining in non-pyknotic nuclei approximately one week before the *Lsd1^{CAGG}* terminal motor phenotype. All IHC (**c-f**) is counterstained with hematoxylin. Scale bars= 50µm.

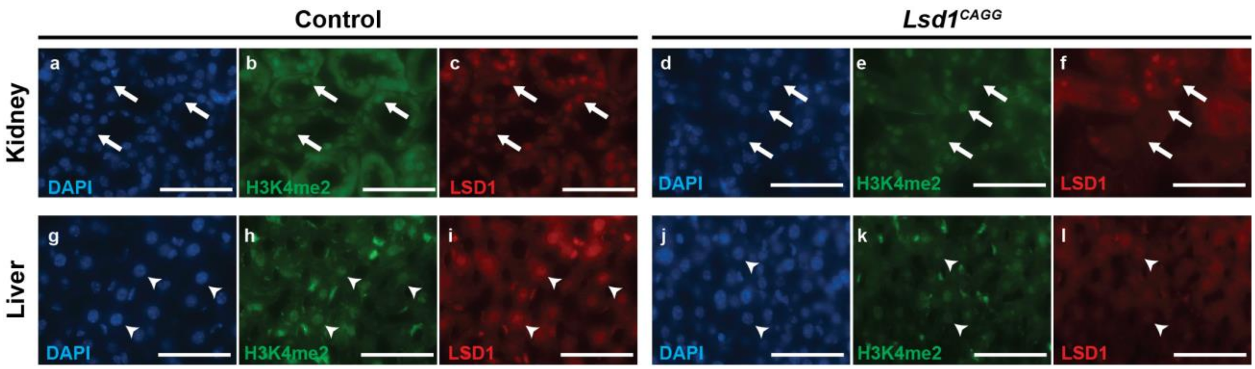

Supplementary Figure 9 | LSD1 is not required for kidney and liver cell viability.

(**a-l**) Representative immunofluorescence images showing LSD1 (red), staining control H3K4me2 (green) and DAPI (blue) in mouse epithelial cells of the kidney nephron (**a-f**, arrows) and hepatocytes of the liver (**g-l**, arrowheads). LSD1 is normally ubiquitously expressed in controls (**c,i**). In *Lsd1*^{CAGG} mice, LSD1 is absent (**f,l**), but kidney and liver morphology remains normal compared to controls (**a,d,g,j**). Absence of LSD1 immunoreactivity is not due to lack of antibody penetrance (**b,e,h,k**). Scale bars= 50µm.

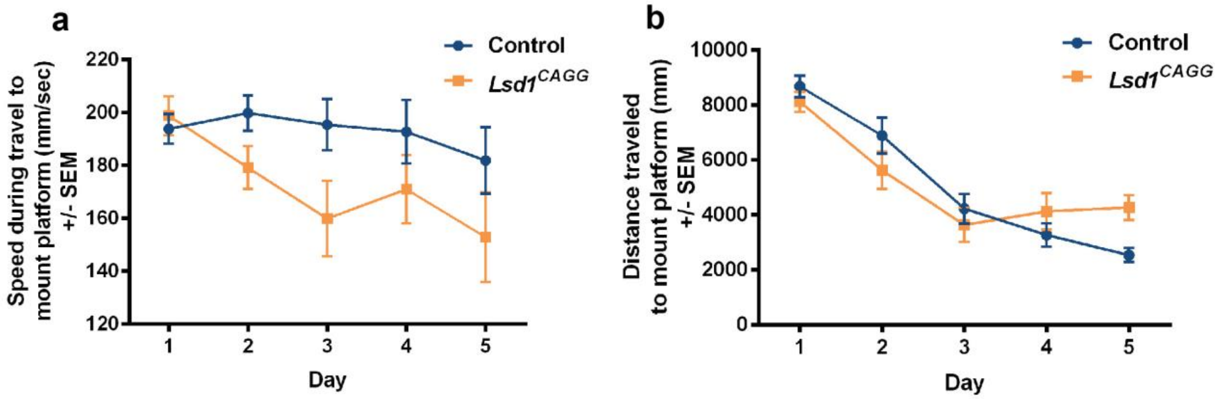

Supplementary Figure 10 | *Lsd1^{CAGG}* mice have learning and memory deficits.

(**a**) Speed during travel to mount platform in Morris water maze across 5 day training period of control (blue, n = 15) and *Lsd1^{CAGG}* (orange, n = 12) mice. Data are shown as mean ± s.e.m. No significant difference between genotypes by repeated measures two-way ANOVA. (**b**) Distance traveled to mount platform in Morris water maze across 5 day training period of control (blue, n = 15) and *Lsd1^{CAGG}* (orange, n = 12) mice. Consistent with the increased latency to mount platform (Fig. 2a), *Lsd1^{CAGG}* mice travel longer distance on Day 5. Data are shown as mean ± s.e.m.

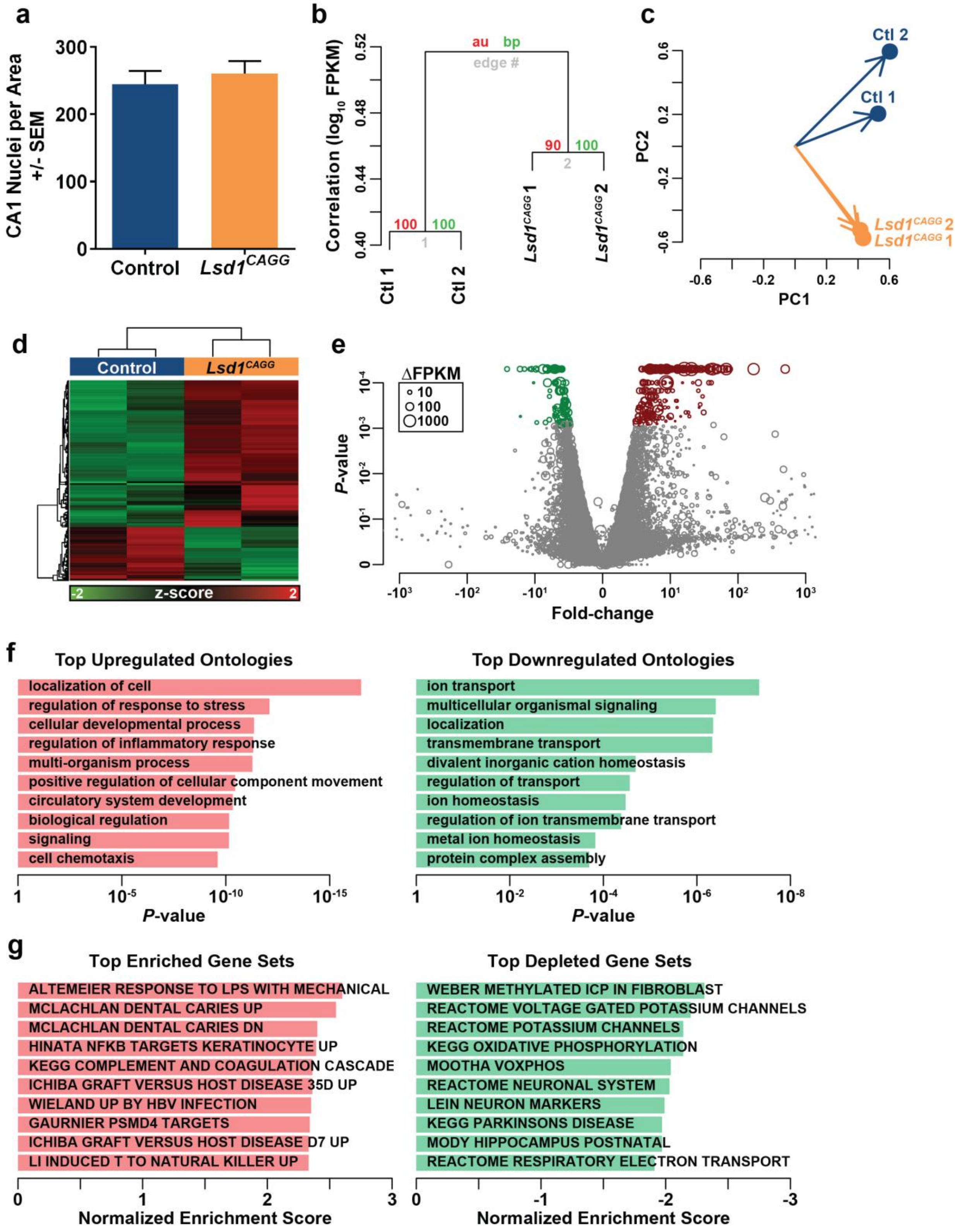

a
CA1 Nuclei per Area +/- SEM
Control
Lsd1^CAGG
b
Correlation (log10 FPKM)
au bp
edge #
Ctl 1
Ctl 2
Lsd1^CAGG 1
Lsd1^CAGG 2
c
PC1
PC2
Ctl 1
Ctl 2
d
Control
Lsd1^CAGG
z-score
e
ΔFPKM
P-value
Fold-change
f
Top Upregulated Ontologies
localization of cell
regulation of response to stress
cellular developmental process
regulation of inflammatory response
multi-organism process
positive regulation of cellular component movement
circulatory system development
biological regulation
signaling
cell chemotaxis
P-value
Top Downregulated Ontologies
ion transport
multicellular organismal signaling
localization
transmembrane transport
divalent inorganic cation homeostasis
regulation of transport
ion homeostasis
regulation of ion transmembrane transport
metal ion homeostasis
protein complex assembly
P-value
g
Top Enriched Gene Sets
ALTEMEIER RESPONSE TO LPS WITH MECHANICAL
MCLACHLAN DENTAL CARIES UP
MCLACHLAN DENTAL CARIES DN
HINATA NFKB TARGETS KERATINOCYTE UP
KEGG COMPLEMENT AND COAGULATION CASCADE
ICHIBA GRAFT VERSUS HOST DISEASE 35D UP
WIELAND UP BY HBV INFECTION
GAURNIER PSMD4 TARGETS
ICHIBA GRAFT VERSUS HOST DISEASE D7 UP
LI INDUCED T TO NATURAL KILLER UP
Normalized Enrichment Score
Top Depleted Gene Sets
WEBER METHYLATED ICP IN FIBROBLAST
REACTOME VOLTAGE GATED POTASSIUM CHANNELS
REACTOME POTASSIUM CHANNELS
KEGG OXIDATIVE PHOSPHORYLATION
MOOTHA VOXPHOS
REACTOME NEURONAL SYSTEM
LEIN NEURON MARKERS
KEGG PARKINSONS DISEASE
MODY HIPPOCAMPUS POSTNATAL
REACTOME RESPIRATORY ELECTRON TRANSPORT
Normalized Enrichment Score

Supplementary Figure 11 | Differential expression of genes in *Lsd1*^{CAGG} hippocampus.

(**a**) Total number of nuclei per area counted in control (n = 4) and terminal *Lsd1*^{CAGG} (n = 10) CA1. Data are shown as mean ± s.e.m. (**b**) Hierarchical clustering of gene expression across 24,412 transcripts (FPKM > 0.5) shows that control and *Lsd1*^{CAGG} replicates significantly segregate by gene expression. The y-axis represents the $\log_{10}$ FPKM correlation. Approximate Unbiased *P*-values (AU, red) and Bootstrap Probabilities (BP, green) for each cluster are shown. (**c**) Principle component analysis (PCA) of 24,412 transcripts (FPKM > 0.5) shows consistent separation of control and *Lsd1*^{CAGG} samples in the first two principle components. (**d**) Heatmap of most significantly differentially expressed (281 upregulated, 124 downregulated) RNA-seq transcripts between *Lsd1*^{CAGG} and control hippocampi. Samples are hierarchically clustered by relative expression of differentially expressed transcripts. Relative higher (red) or lower (green) expression is indicated. (**e**) Volcano plot of fold-changes in gene expression (x-axis) by statistical significance (*P*-value; y-axis). Each circle represents a transcript and the normalized change in expression is represented by the size of the circle (legend). Those transcripts that are significantly (FDR < 0.05) differentially expressed are represented in red (281 upregulated) and green (124 downregulated). (**f**) Histogram of Gene Ontology analysis shows ontologies that are associated with those genes that are upregulated (red) and those genes that are downregulated (green) in the *Lsd1*^{CAGG} RNA-seq dataset. The top 10 ontologies are shown with P-values. (**g**) Histogram of Gene Set Enrichment Analysis shows the most enriched (red) and depleted (green) gene sets in the *Lsd1*^{CAGG} RNA-seq dataset. The top 10 gene sets are shown with normalized enrichment scores.

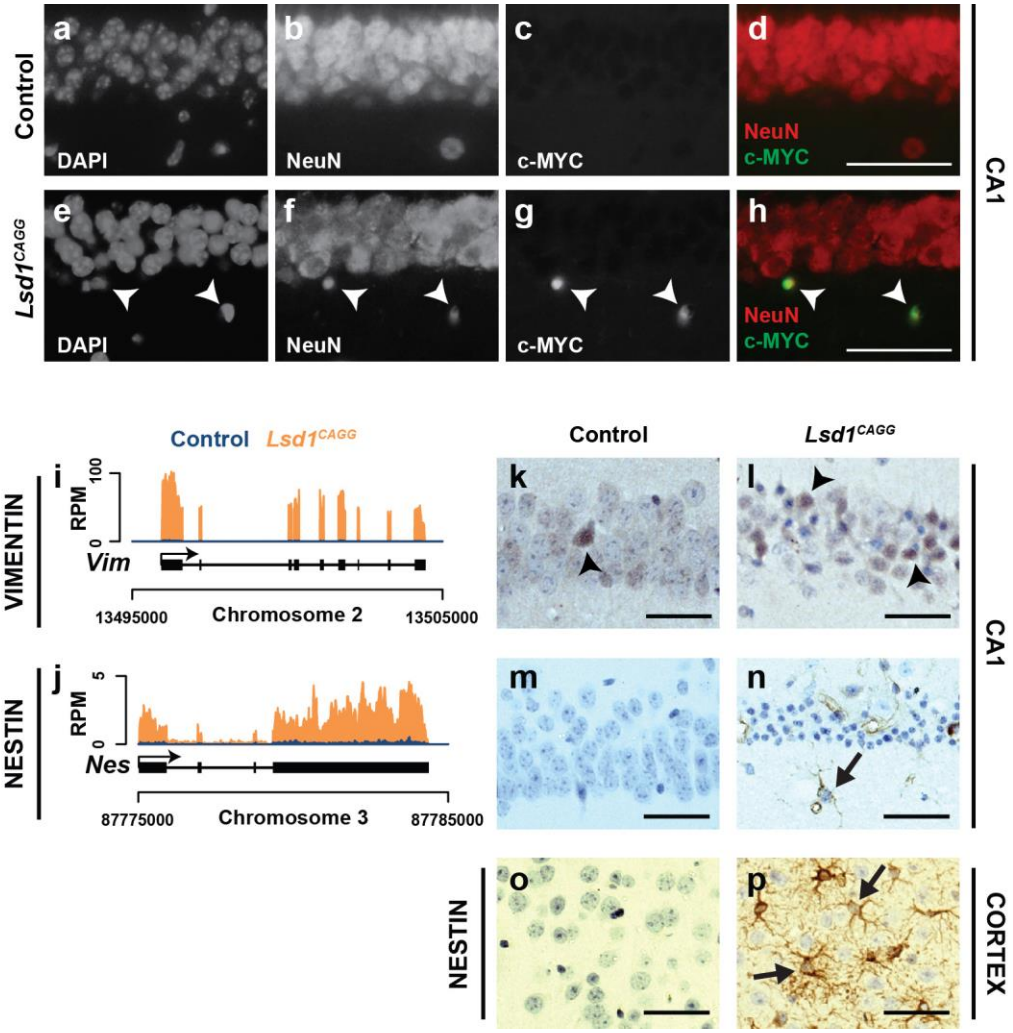

Supplementary Figure 12 | Neural stem cell gene expression in *Lsd1*^{CAGG} mice.

(**a-h**) Immunofluorescence labelling of DAPI (**a,e**), NeuN (**b,f**), c-MYC (**c,g**) and NeuN/c-MYC merge (**d,h**) in control (**a-d**) and *Lsd1*^{CAGG} (**e-h**) CA1. c-MYC protein is present in the nuclei of neurons in *Lsd1*^{CAGG} mice (**e-h,** arrowheads), but absent from neurons in control mince. (**i,j**) Genome browser style plot of RNA-seq reads per million (RPM) from control (blue) and overlaid *Lsd1*^{CAGG} (orange) hippocampus showing expression of the genes *Vimentin* (**i**) and *Nestin* (**j**). (**k-p**) Immunohistochemistry (IHC) with antibodies to VIMENTIN (**k,l**) and NESTIN (**m-p**) in control CA1 (**k,m**) and cortex (**o**), and *Lsd1*^{CAGG} CA1 (**l,n**) and cortex (**p**). VIMENTIN immunoreactivity was present in CA1 neurons in both control (**k**, arrowheads) and *Lsd1*^{CAGG} (**l,**

arrowheads), with more immunoreactive neurons in *Lsd1*^{CAGG}. NESTIN immunoreactivity was found in glial-shaped cells in *Lsd1*^{CAGG} hippocampus (**n**) and cortex (**p**, arrows) and absent in control (**m,o**). All IHC is counterstained with hematoxylin. All *Lsd1*^{CAGG} images were taken at the terminal phenotype. Scale bars= 50µm.

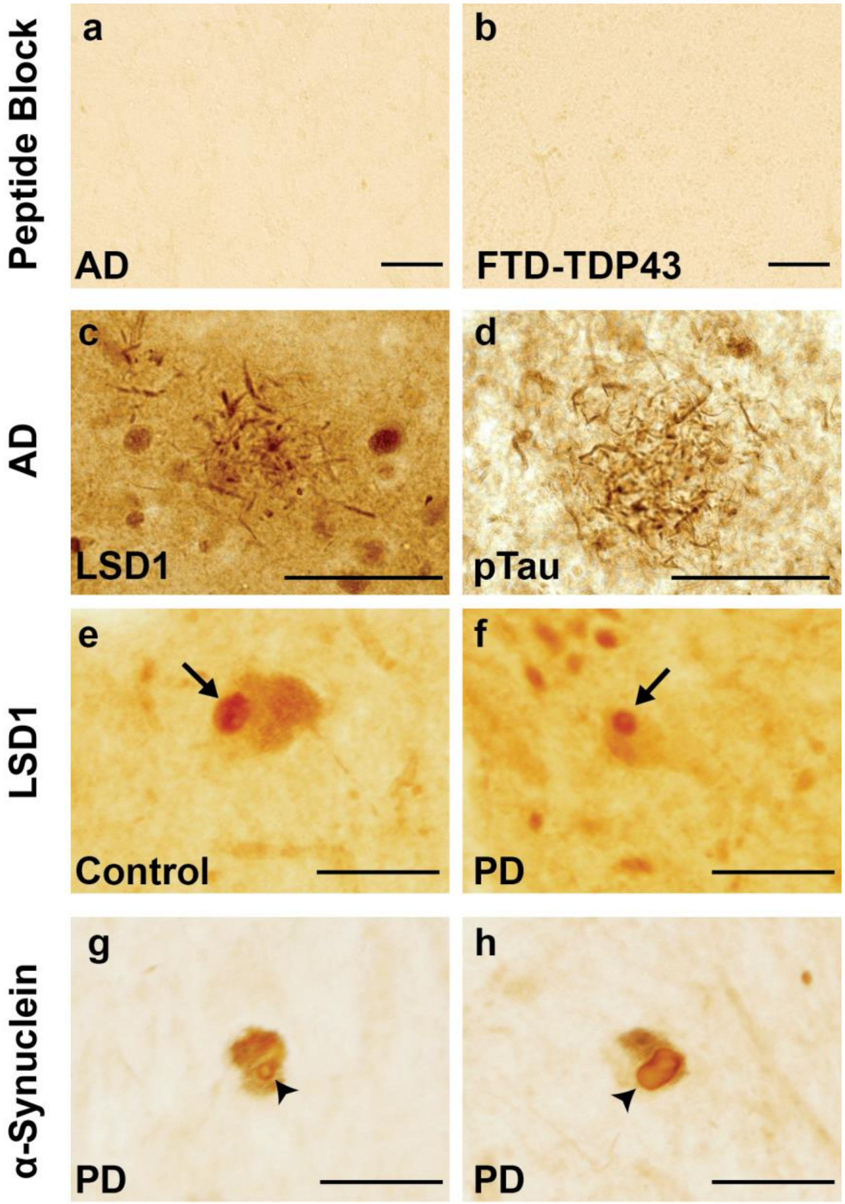

Supplementary Figure 13 | LSD1 mislocalization is specific to AD and FTD.

(**a,b**) LSD1 IHC with primary antibody preincubated with the target peptide shows an absence of signal in AD (**a**) and FTD-TDP43 (**b**). (**c,d**) LSD1 (**c**) and pTau (AT8 epitope) (**d**) immunohistochemistry (IHC) showing immunoreactivity localized to neurites (**c**) and neuropil threads (**d**) around a senile plaque, but not to the amyloid core of the plaque. (**e,f**) LSD1 IHC in control (**e**) and PD (**f**) dopaminergic neurons of the substantia nigra shows LSD1 localized to the

nucleus (arrows) and not Lewy bodies. (**g,h**) α-Synuclein IHC in PD shows formation of Lewy bodies in dopaminergic neurons of the substantia nigra (arrowheads). Scale bars= 50µm.

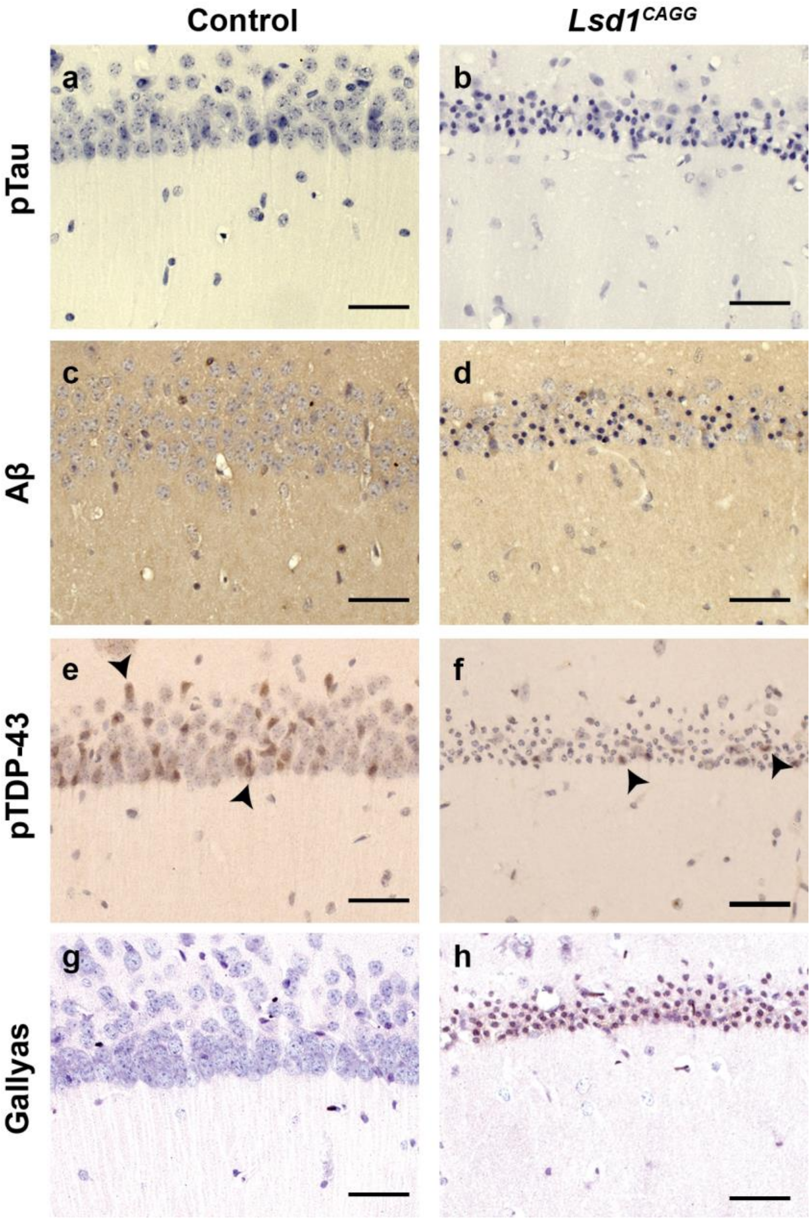

Supplementary Figure 14 | Absence of pathological protein aggregates in *Lsd1*^{CAGG} mice.

(**a-f**) pTau (AT8 epitope) (**a,b**), Aβ (**c,d**), and pTDP-43 (**e,f**) immunohistochemistry in control (**a,c,e**) and *Lsd1*^{CAGG} (**b,d,f**) CA1 neurons showing absence of aggregate forms of the proteins. pTDP-43 is found sporadically in control nuclei (**e**, arrowheads) and shows a similar staining pattern in *Lsd1*^{CAGG} non-pyknotic nuclei (**f**, arrowheads), but there is no evidence of pTDP-43

aggregation. (**g,h**) Gallyas silver staining in control (**g**) and *Lsd1*^{CAGG} (**h**) CA1 neurons showing lack of any protein aggregation (positive stain is black). Scale bars= 50μm.

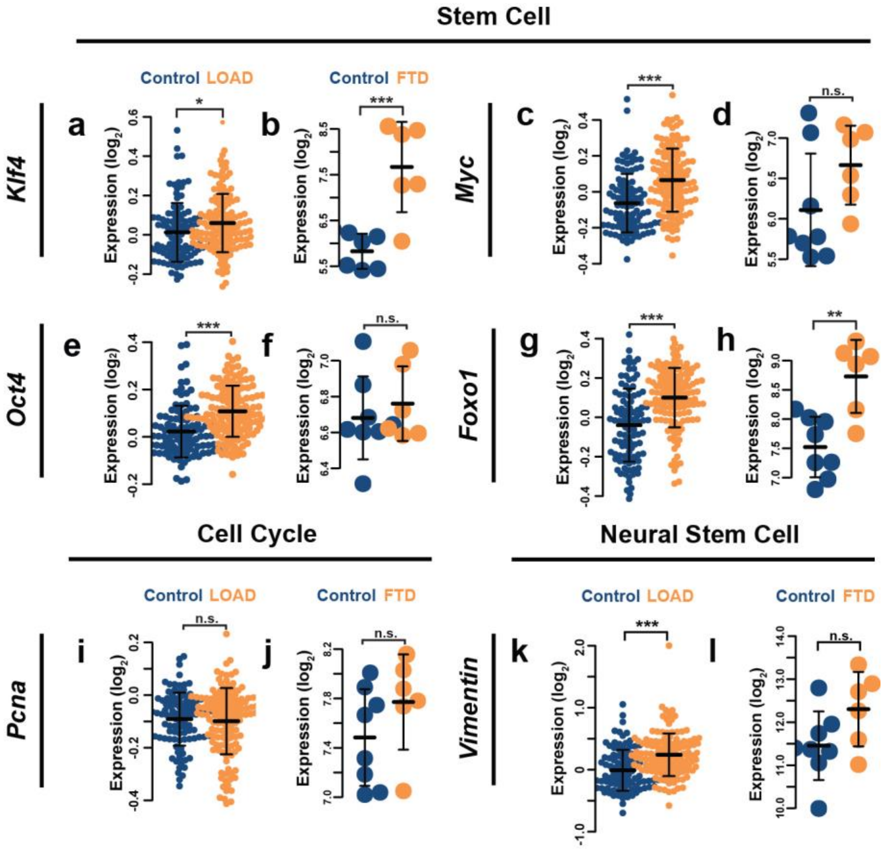

Supplementary Figure 15 | Stem cell gene expression in human dementia.

(**a-n**) Beeswarm plots showing expression of *Klf4* (**a,b**), *Myc* (**c,d**), *Oct4* (**e,f**), *Foxo1* (**g,h**), *PCNA* (**i,j**) and *Vimentin* (**k,l**) in control (blue) versus LOAD prefrontal cortex[29] (**a,c,e,g,i,k,** orange), or control (blue) versus FTD-progranulin frontal cortex[30] (**b,d,f,h,j,l,** orange). Values represent the $\log_2$ expression of each patient and bars represent mean ± s.d., $*P < 0.05$, $**P < 0.01$, $***P < 0.001$, n.s.: not significant.

Target	Manufacturer	Experiment	Dilution
NeuN	Millipore MAB377	Mouse IF	1:100
LSD1	Abcam 17721	Mouse IF	1:200
		Human IHC	1:500
		Human IF	1:500
		Mouse IHC	1:500
pTau (AT8 epitope)	ThermoFisher MN 1020	Human IHC	1:1,000
		Human IF	1:1,000
		Mouse IHC	1:1,000
pTDP-43	Cosmo Bio TIP-PTD-P02	Human IHC	1:1,000
		Mouse IHC	1:4,000
pTDP-43	Cosmo Bio TIP-PTD-M01	Human IF	1:1,000
α-Synuclein	J. Trojanowski and V. Lee Labs	Human IHC	1:10,000
Map2	Chemicon AB5622	Mouse IHC	1:500
Tau	Accurate BYA10741	Mouse IHC	1:200
GFAP	Dako Z0334	Mouse IHC	1:100
SV2	DSHB SV2	Mouse IF	1:50
Aβ	Signet 9220-02	Mouse IHC	1:1,000
KLF4	R&D Systems AF3158	Mouse IHC	1:100
c-MYC	Santa Cruz SC-40	Mouse IHC	1:100
OCT-4	BD Transduction Labs 611202	Mouse IHC	1:300
FOXO-1	Santa Cruz SC-11350	Mouse IHC	1:100
PCNA	Santa Cruz SC-56	Mouse IHC	1:250
H3S10p	Active Motif 39254	Mouse IHC	1:1000
NESTIN	Abcam ab11306	Mouse IHC	1:1000
VIMENTIN	Dako M072529-2	Mouse IHC	1:50
MBP	Millipore MAB386	Mouse IHC	1:100
CC1	Millapore OP80	Mouse IF	1:20
IBA1	ThermoFisher MA5-15810	Mouse IF	1:500

Supplementary Table 1 | Primary antibodies used for immunohistochemistry (IHC) and immunofluorescence (IF) experiments.

Shown for each antibody are the target antigen, manufacturer, experiments used and corresponding experimental dilution.
